# Notes and News

**Published:** 1890-02-01

**Authors:** 


### Notes and News.

Collected and Communicated.

The Jenny Lind Infirmary for sick children at Norwich is an institution which always appeals to the sentiment. Still it has to report a falling off in annual subscriptions, and our artistic friends had better come forward in the interests of this infirmary. Over 240 in-patients and 1,230 out-patients were treated last year, and so successfully that though 118 operations were performed they only resulted in one death. Amongst those present at the annual meeting were the Mayor, the Bishop and the Dean of the town, Lady Eade, Sir Harry and Lady Bullard, Dr. Bunnett, and Colonel Winter.

Chester Hospital Saturday Committee work well. The following figures will show what has been done since the movement was started: 1886, £136 9s. 8½d.; 1887, £209 1s. 10¼d.; 1888, £291 16s. 0¼d.; 1889, £300 11s. 10d. The following resolution has just been brought before the Hospital Committee: "That, in consideration of nearly £1,000 having been collected for the infirmary by the Chester and District Working Men's Hospital Saturday Committee during the past four years, a governorship be granted for each £20 collected on behalf of the infirmary, and that the committee elect their own governors according to their subscription list each year." Amongst the collection this year was one of £5 from the women of the Chester Steam Laundry.

Barnet Cottage Hospital has justified its existence. In the first report, just published, the following particulars of cases treated are given, and surely such cases could only be properly treated in a hospital. During the year, 76 patients were admitted as in-patients to the hospital; of these, 18 were medical and 58 were surgical cases. There were eight fractures treated—viz., two of fibula, one humerus, one radius, one ribs, three compound fractures of skull, and one compound dislocation of ankle. Twenty operations were performed in the year, with only two deaths—viz., one from strangulated hernia, and one compound fracture of skull. In all, there were eight deaths —viz., one fracture of skull (died five minutes after being admitted), one diabetes, one compound fracture of skull after operation), one meningitis, and three heart disease.

The annual report of Lyme Regis Cottage Hospital tells of 46 in-patients treated at a total expenditure of £235. This is good. The hospital is to be enlarged by a lean-to bathroom of wood and felt. The new matron, Miss Carter, not only does all the nursing of the hospital, but attends such dispensary cases as need her care.

Pendlebury Children's Hospital treated 924 in-patients last year. In the medical report the subject of the insurance of very young children was once again dealt with, and the facts stated could not fail to arrest attention. The comment of the medical men, based upon an additional year's experience, that further legislation was required called for attention, as their practical work entitled them to speak with more than ordinary authority. The fund for sending children to the seaside, originated by Mr. William Agnew, is doing good work and flourishing. Mr. and Mrs. Agnew have endowed another cot.

The conference on hospital abuse at Birmingham on the 15th did not result in any elucidation of the problem how to separate the sheep from the goats; for the medical men refused to bring forward any definite scheme. So ultimately it was resolved that a committee of laymen—that is, of hospital managers who are not medical men—should be appointed, that some medical men should be added to it, and that there should be "a chairman not interested in the question," rather a difficult person to be found. The Birmingham papers have taken up the subject with enthusiasm, and have published long articles explaining the Manchester and other systems of out-patient relief, so that the conference could not plead ignorance on the subject it met to discuss.

The influenza is still with us; like some mischievous sprite it is now here, now there, in the castle or in the garret, in the crowded town or the scattered village. At Vienna and at Brussels they think they have discovered the microbe of the disease. Dr. Symes Thompson has delivered a series of lectures on the influenza, and told us much that is interesting and historical concerning it, but he did not venture to become too definite as to its origin or the proper treatment. You cannot take liberties with the influenza, he is too lively. He resents all questions as to his parentage, and flits away to fresh fields as soon as the medical officer of health begins to enter particulars concerning him in a note-book. Let us hope the Vienna doctors have found the microbe, but let us hope they will not want to inoculate us with it.

It is a dreadful point about these microbes that the only way to avoid having them in a virulent form is to have them in an artificial or attenuated form. The children of the future will not run through the present gamut of infantile disease, but they will probably be subjected to inoculation with various microbes every few months. First, they will be vaccinated for smallpox; when they have recovered from that, they will be taken to a Pasteur institute to have a mild form of rabies. Next, they will be given a dose of the comma bacilli to prevent cholera, and so on through all the ever-growing series of disease microbes. Oh! luckless child of the future! you will never be ill and never be well; your health will be awfully monotonous; you will never know the weariness of the first night of measles, when it was so nice to lie in mother's lap and feel her cool hand on your forehead; you will never know the joys of convalescence, when oranges were numerous and everyone was kind to you because you were not well; and your end will be to die of debility. How glad we are that we live in the present, with all its ups and downs of health to lend variety to life and death!

The following vacancies are announced this week, the amount of the salary in each case being given after the name of the institution:

**Resident Medical Officers.**

Victoria Park Chest Hospital. House Physician—board, &c.
Bristol Hospital for Children. House Surgeon—£100, board, &c.

**Non-Resident Medical Officers.**

London Hospital. Curator's Assistant—£52.

West End Hospital. Dental Surgeon.

Westminster Dispensary. Honorary Physician.

---

THE Hospital Saturday Fund delegates held a meeting on Saturday in Mitre-court, Temple. The secretary reported that £14,000 had been collected during the past year, against £11,000 the previous year. Subscriptions to the amount of £1,360 had been received up to Friday night on account of the current year.

---

THE report of Canterbury Dispensary is out. During the year there have been admitted 1,757 patients, an increase of about 50 over the preceding year; 1,449 of these have been attended at their own homes, 1,245 have been cured, 303 were relieved, 58 died, and 145, at the remainder of the year, were under treatment. The financial statement showed that £4,309 12s. 1d. was invested as capital. The receipts for the year amounted to £565 8s. 5d. The dispensary has existed for over 50 years, and has always managed to pay its way.

---

DURING the fourteen years that the Jessop Hospital, Sheffield, has been opened neither concert nor amusement of any kind has been allowed either for patients or nurses until the 15th January, when the present matron and house surgeon had permission from the board and staff to give an entertainment. The evening commenced with an excellent concert, waxworks following, Dr. J. A. Hall (late of Bartholomew's) taking the principal part in a most creditable manner. Refreshments were served to almost 100 patients and visitors, and a dance ended a very successful evening.

---

SUCH a pretty programme from Rotherham of the hospital entertainment there! The patients also seem to have had liberal fare, and the matron and nurses did all they could to make the festive season as happy as possible. The provident members of Boscombe Hospital gave their entertainment on January 13th. There was a magic lantern, a concert, and a Christmas tree, all under the direction of the matron, Miss Fletcher, and all the provident members were most pleased with the gifts they received. With these two late notices we really must bid farewell to Christmas, and return to the sober study of annual reports and the cost per patient per day.

---

IN connection with Salisbury Provident Dispensary is a loan fund, which might well be added to other dispensaries. At the annual meeting Dr. Roberts said with regard to this fund: "When some misfortune occurred, and help was required, a petition was drawn up, and armed with this document, people went about begging. In this way they soon obtained the sum they required without the bother of having to find sureties and without the unpleasantness of having to pay the money back. He was afraid they must come to the conclusion that to beg they were not ashamed, for it had become a general custom which tended to destroy—and, he believed, often did destroy—all idea of self-dependence and self-respect. The object of the loan fund was to try and check, in some small degree, this unhappy custom, and to save a few from sinking to the degradation of beggary."

THE following is the substance of the report on the accounts of the St. John's Hospital for Skin by Messrs. W. H. Pannell and Co., chartered accountants. It is dated 3rd January, from Library-chambers, 13, 14, Basinghall-street, E.C. We deal with it on page 286:

We have examined the books of St. John's Hospital for Diseases of the Skin, in accordance with the instructions of the Hospital Saturday Delegates, which were (1) to satisfy ourselves that all the receipts had been properly accounted for during the past two years; (2) to compare the returns made to the fund by the hospital for the year 1887, and the fifteen months from the 1st January, 1888, to 31st March, 1889, with the latter's books; and (3) to give our opinion of the financial management of the hospital. We remind you that you did not wish us to go over the ground covered by the judicial investigations.

BOOKS.

Primarily, we would remark that the system of book-keeping at the hospital is very cumbersome, as the receipts and payments are entered in the same book as that in which the tradesmen's weekly accounts appear; these would be much better entered in a separate book. The cash-book having been filled up in August, the record of receipts, payments, etc., is now being written upon loose sheets of paper kept in the cash-book; these we understand are to be incorporated therewith at the end of the year. Mr. St. V. Mercier admits this is being done against the desire of Mr. Lemon.

RECEIPTS.

Under the first of the above heads we have to report as follows: We have examined the whole of the counterfoils of the receipt-books in which the amounts vary, and have traced the sequence of those receipt-books which are printed for special amounts. The cash has all been traced into the bank or properly accounted for; there are a few inaccuracies in the figures, but of trifling amounts; in the earlier part of the period we are dealing with, there were several instances of delay in the payments into the bank, but since July last, as above mentioned, there is no cause for complaint on this ground.

PAYMENTS.

The payments are fairly well supported by vouchers, and we take no exception to these other than those hereafter mentioned in this report.

GENERALLY.

In March last, on the closing of the books, an amount of £6 10s. 10d was overpaid in the petty cash. This was brought into the general cash-book, and so decreased the balance in hand shown by that book; in the petty cash-book, however, the amount was brought forward as due to Mr. Mercier, and has thus been included in the payments twice, and we have now made the necessary correction in the petty cash-book. We fail to understand how this could have escaped the attention of an auditor.

RETURNS.

Under the second head we have to report as follows: On comparing the returns made to your board by the hospital with their books, we find the following worthy of notice:

| | | 1887. 12 Months. | | 1888-9. 15 Months. |
|---|---|---|---|---|
| The returns say | Donations.. | £998 14 6 | .... | £765 10 6 |
| The books show for the same period | Donations........ ..... | £118 6 0 | .... | £48 4 0 |
| | Voluntary subscriptions | *870 12 0 | .... | 717 6 6 |
| | Advertising............ | 9 16 6 | .... | — |
| | | £998 14 6 | | £765 10 6 |

*These amounts are made up of sums of 1s., 2s., or 3s. contributed by the out-patients alone, and not by the public, the amount entered in the returns to you as received from out-patients being regularly instalments of 3s., 5s., or 10s. per month.

The number of attendances of patients mentioned in the returns is not an ascertained number, but is arrived at in the following manner: To the total number of free tickets issued to patients is added four times the number of paying tickets issued; each week's medicine supplied is thus considered as an attendance. Thus if a patient (as is often the case) has medicine for four or eight weeks given him at the first attendance, this would be counted as four or eight attendances.

SECRETARY'S SALARY.

And, thirdly, we have to report as follows: The secretary appears to have been paid £150 per annum and commission of 15 per cent. upon the receipts after the first £1,000, the total remuneration not to exceed £325; but in January, 1889, the board fixed the salary at this full sum without commission. We are unable to make the amount drawn by Mr. St. V. Mercier before January, 1889, agree with the salary and commission payable to him, but he does not seem to have exceeded the amount so payable. In addition to this salary, the secretary for the time being was entitled, by a minute of the board in November, 1876, to have certain meals in the hospital, and it seems that when Mr. Mercier was away from the hospital on its business, or when such meals could not be provided, he drew 1s. 6d. or 1s. 9d. a day in lieu thereof. This was stopped in July, 1889, when the board decided such amounts should only be allowed in future on very good cause being shown.

We understand that cheques are signed by Col. Mercier and Mr. Mercier jointly, and we think it desirable that there should be a countersignature of one of the members of the board as evidence that the payment is authorised by the board.

The system of daily payments into the bank now in use is satisfactory, and prevents (while strictly adhered to) the reoccurrence of the former irregularities.

